# Defining the early systemic neutrophil signature after severe trauma: decreased CD10/CD16 levels on admission precedes metabolic dysregulation

**DOI:** 10.1007/s00068-025-02980-x

**Published:** 2025-11-14

**Authors:** Christian Thomas Hübner, Emma de Fraiture, Roman Pfeifer, Nathalie Piaz, Felix Karl-Ludwig Klingebiel, Yannik Kalbas, Paolo Cinelli, Leo Koenderman, Falco Hietbrink, Hans-Christoph Pape, Michel Paul Johan Teuben

**Affiliations:** 1 Department of Traumatology, University Hospital Zurich, University of Zurich, Raemistrasse 100, 8091 Zurich, Switzerland; 2 Department of Trauma Surgery, University Medical Center Utrecht, Utrecht, The Netherlands; 3 Center for Translational Immunology (CTI), University Medical Center Utrecht, Utrecht, The Netherlands; 4 Department of Respiratory Medicine, University Medical Center Utrecht, Utrecht, The Netherlands

**Keywords:** Polytrauma, Critically ill patient, Neutrophil, Injury, Trauma, Infection

## Abstract

**Purpose:**

Dysregulation of the post-traumatic immune response forms the basis for life threatening complications such as acute respiratory distress syndrome and (multiple) organ failure. Automated flowcytometry analysis of peripheral blood samples enables rapid immunomonitoring in the shock room. It was hypothesized that a specific systemic neutrophil receptor expression pattern in peripheral blood is associated with early metabolic deterioration, as indicated by high lactate levels after eight hours in severely injured trauma patients.

**Methods:**

Severely injured trauma patients with an Injury Severity Score > 25 and healthy volunteers, were prospectively included in a Level I trauma center in Central Europe. The systemic neutrophil receptor signature in peripheral blood was determined by measuring the neutrophil markers CD16, and CD10 with an AQUIOS CL^®^ “load & go” flow cytometer.

**Results:**

A total of 24 patients with a mean age of 51.5 years and a median ISS of 36.5 were included, as well as a control group of 9 healthy volunteers. Mean admission leukocyte levels of 15.2 G/L were seen. Circulatory leukocyte counts were significantly higher and cell surface expression levels of CD10 und CD16 were significant lower in trauma patients compared to healthy controls. Spearman’s rank correlation coefficients for CD10 (ρ = -0.649) and CD16 (ρ = -0.493) showed significant negative correlations with elevated lactate levels after 8 h.

**Conclusion:**

This study shows that admission CD10 and CD16 expression levels on neutrophils correlate with elevated lactate levels 8 h post-injury in severe polytrauma. A specific CD10/CD16 neutrophil signature, linked to leukocytosis, precedes metabolic dysregulation, determined by higher lactate levels. A better understanding of the early neutrophil receptor profile may form the basis for future immunomonitoring and guide treatment decisions.

## Introduction

Dysregulation of the post-traumatic immune response forms the basis for life-threatening inflammatory complications such as Acute Respiratory Distress Syndrome, sepsis and Multiple Organ Dysfunction Syndrome (MODS) [1, 2]. Neutrophils are a key element of the innate cellular immune system and are the first cells responding to injury [3]. In addition to the activation and extravasation of neutrophils, the mobilization of neutrophils from the bone marrow has also been described after trauma [4]. Changes in neutrophil compartmentalization likely affect the transition from regular, non-harmful, and balanced inflammatory responses into exaggerated pathological responses. Alterations in the presence of neutrophil subtypes in circulation can be monitored by measuring variations in the expression of cell surface markers by systemic neutrophils after trauma. In order to monitor shifts in the systemic neutrophil pool, well-described maturation markers CD10 and CD16 are of particular interest. This is especially important because low overall expression of these markers is associated with the presence of immature neutrophils in the circulation. In the case of systemic inflammation this reflects the release of immature cells from the bone marrow into circulation [5, 6]. Early post-traumatic alterations in the composition of the circulatory neutrophil pool have been linked to impaired outcome [7].

In the current study it was hypothesized that early decreased levels of CD10/CD16 on systemic neutrophils on admission are associated with elevated lactate levels 8 h post-injury in severely injured polytrauma patients.

## Methods

The study has been performed at a Level I trauma center in a metropolitan area in Central Europe. The project was approved by the local ethical commission (BASEC 2017 − 01380). Patient consent was obtained according to the study protocol in accordance with local legal regulations, international ethical standards and the Declaration of Helsinki.

Severely injured trauma patients were included if they met the following inclusion criteria:

- Injury Severity Score (ISS) ≥ 25.
- age > 18 Years.
- agreement after informed consent.

Exclusion criteria included:

- transferred patients.
- dead on arrival.
- Flawed sampling for neutrophil analysis.
- Immune-deficiency due to medical condition or medical treatment.
- pregnancy.

*Outcome parameters included:*

- Circulatory leukocyte numbers on admission.
- Blood neutrophil cell surface expression levels of CD10 on admission.
- Blood neutrophil cell surface expression levels of CD16 on admission.
- Lactate levels 8 h after insult to monitor metabolic status.
- Occurrence of infectious complications.

Patient related parameters included: Age, sex, BMI. Trauma related factors: Systolic Arterial Pressure, Mean Arterial Pressure (MAP), Heart Rate (HR), Glasgow coma scale (GCS). Standard laboratory parameters: Leukocyte counts, Serum Hemoglobin (g/l), differential blood count, Quick, INR, CK. ISS [8] was calculated using the abbreviated injury scale 2008 [9]. In patients, blood for flow studies was drawn with routine blood collection immediately after arrival in trauma bay, sampling occurred within one hour. Average time from trauma to arrival in trauma bay is one hour.

### Control group of healthy volunteers

In order to set baseline levels of neutrophil cell-surface expression levels, a group of 9 healthy volunteers (5 female, 4 male) with an average age of 26.7 years was sampled alongside with the severely injured trauma patients. After obtaining informed consent and performing peripheral venous punction, blood was immediately proceeded to below mentioned protocol. Thereby, physiological neutrophil expression levels were determined and this enabled a comparison with the trauma cohort.

### Sampling and laboratory protocol

Blood samples for flowcytometry measurements were collected and analyzed using automated AQUIOS CL^®^ “load & go” flow cytometer. The protocol has been described by others in detail [10]. In short, blood collection tube was placed into the device and blood is pipetted automated to a 96-well plate. First a staining step with an 18-µL customized antibody mixture that contains CD16-FITC (FcgRIII/clone 3G8) and CD10-PC5 (neutral endopeptidase/clone ALB1), among others was conducted (both antibodies manufactured by Beckman Coulter Life Science, Krefeld, Germany). Next step was the lysing reaction of red blood-cells using 335 µL AQUIOS Lysing Reagent A (a cyanide-free lytic). Lysis is terminated by pipetting 100 µL of AQUIOS Lysing Reagent B into the well. Finally, the reagent was analyzed in the flow-cell. Data were exported as FCS 3.1 High Res Listmode Files (.lmd) and imported to FlowJo (De Novo Software, Glendale, CA, USA, Version 10.10.0). Gating of neutrophil and eosinophil granulocytes was done as previously validated and described by Spijkerman et al. [11] by forward-sideward scatter after the exclusion of debris and doublet cells. There was no need to add viability stains as pilot and validation experiments have demonstrated an > 99% viability level of fresh samples. Given these high viability-rates, no cell-fixation step was included in the protocol. All samples have been analyzed within 30 min after collection. A minimum of > 100,000 cells per sample has been defined for the determination of median fluorescence intensity (MFI)-levels. The markers were analyzed and compared by calculating the MFI.

Blood for the determination of serum lactate level has been collected eight hours after trauma. Analysis has been performed by blood gas analysis (ABL800 Flex, Radiometer, Thalwil, Switzerland) and a total of 2.5 ml of blood was used and stored in.

Values were exported and statistical analysis was performed using R-Studio (R Foundation for Statistical Computing, Vienna, Austria, Version 2024.12.1 + 563). For comparison of both groups a two-sided Mann-Whitney-Wilcox-test with a significance level of 0.05 was used. Spearman’s rank-correlation and Pearson correlation coefficient were used for correlation analysis. Evans-classification was used to determine the level of correlation [12].

## Results

A total of 26 patients were included in the shock room that met the inclusion criteria including the expectation of an ISS of > 25. Nine healthy volunteers were measured as controls. One patient was excluded from the study since the final ISS after the complete diagnostic evaluation was 21. Another patient was excluded, because no 8 h-lactate measurement was performed.

Among the 24 included patients, 14 were male and 10 were female. Mean age of the patients was 51.5 (± 18.9) years and a median ISS of 36.5 [29–43] was calculated. A median Glasgow Coma Scale (GCS) of 12.5 [7.75–14.25] was reported at scene. Regarding hemodynamic parameters, the systolic arterial blood pressure and mean arterial pressure were 123 mmHg (± 31.7) and 74.1 mmHg (± 39.9), respectively. The average heart rate (HR) was 90.2 bpm (± 14.9). Mean admission leukocyte levels of 15.2G/l (± 5.8) were seen. Table 1 shows an overview of patient and trauma characteristics.

**Table 1:** Clinical baseline characters

Mean age in years (Range)	51.5	(19.0–83.0)
Sex *n* (%)		
Female	10	(41.7)
Male	14	(58.3)
Mean vital parameters (SD)		
Systolic arterial pressure (mmHg)	123.0	(31.7)
MAP (mmHg)	74.1	(39.9)
HR (bpm)	90.2	(14.9)
Mean laboratory parameters (SD)		
Serum Hemoglobin (g/L)	118.9	(21.4)
WBC (G/L)	15.2	(5.8)
Neutrophils	12.2	(5.2)
Monocytes	0.8	(0.4)
Eosinophils	0.1	(0.1)
Quick	75.7	(29)
INR	1.21	(0.50)
CK	520.13	(503.72)
pH	7.332	(0.057)
Base Excess	−3.4	(2.4)
Median GCS (Range)	12.5	(3–15)
Median ISS (Range)	36.5	(25–48)
Trauma mechanism n(%)		
Explosion	1	(4.2)
Fall	9	(37.5)
Gun shot incident	2	(8.3)
Traffic accident	8	(33.3)
Other	4	(16.7)

### Blood neutrophil expression of CD10 and CD16: polytrauma vs. healthy control

**Fig. 1:**
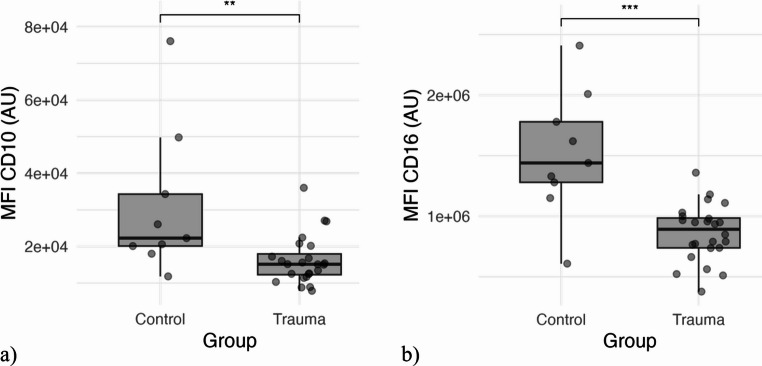
**a**) Boxplot-diagram comparing expression of CD10 in trauma and control group. **b**) Boxplot-diagram comparing expression of CD16 in trauma and controlgroup

In polytraumatized patients, neutrophil CD16 expression levels were significantly lower compared to healthy controls (*p* < 0.001). More specifically, CD16 expression on circulatory neutrophils in the trauma group was 57% of baseline levels measured in the control group (Fig. 1b). A similar observation was made for neutrophil CD10 expression, where the mean expression-level was significant lower, than in the control-group (*p* = 0.007). A boxplot of the CD10 cell surface expression levels in severely injured polytraumatized patients and control group, is demonstrated in Fig. 1a. In the trauma group a subpopulation of 44.7% (± 17.2%) CD10^low^ and 30.3% (± 13.6%) CD16^low^ was observed.

### Correlation of circulatory neutrophil CD10 and CD16 with blood lactate levels

**Fig. 2:**
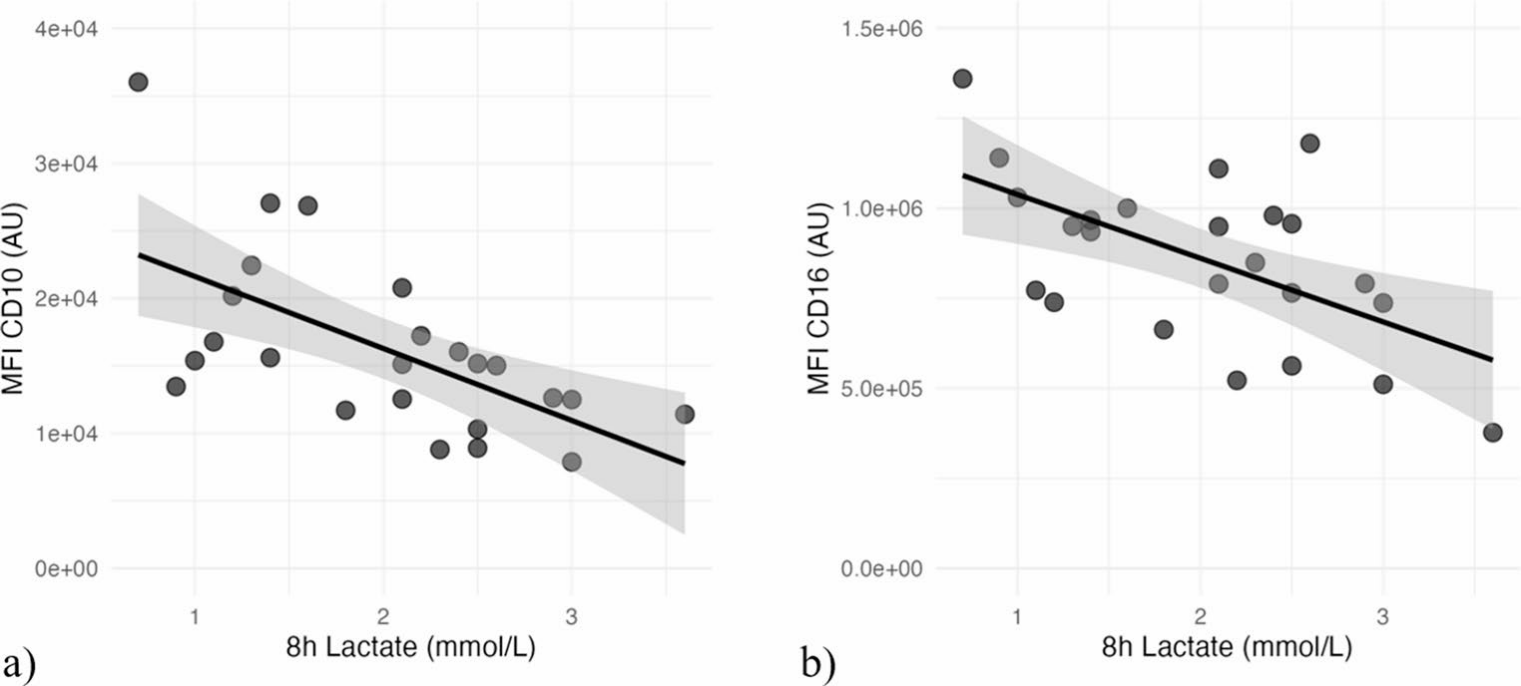
**a**) Diagram with correlation between 8h lactate (mmol/l) and MFI CD10. **b**) Diagram with correlation between 8h lactate (mmol/l) and MFI CD16

The 8 h lactate levels ranged from 0.7 to 3.6 mmol/L, with a mean of 2.01 mmol/L and a standard deviation of 0.77 mmol/L. Aberrant cell surface expression levels of CD10 and CD16 on systemic neutrophils correlated with the 8 h lactate levels. The correlation between CD10 and 8 h lactate levels was characterized by a Spearman’s rank correlation coefficient of −0.649 (*p* < 0.001) resulting in a strong negative correlation (Pearson`s *r* = 0.619) (Fig. 2a) and for CD16 a moderate negative correlation was seen (Spearman’s rank correlation coefficient (ρ)=−0.493, *p* = 0.01, Pearson`s *r* = 0.582) (Fig. 2b). The percentage of CD10^low^ correlated moderate (ρ = 0.419, *p* = 0. 07) and the percentage of CD16^low^ very weak (ρ = 0.107, *p* = 0.65). However, these findings were not significant.

### CD10 and CD16 expression levels in patients with infectious complications

**Fig. 3:**
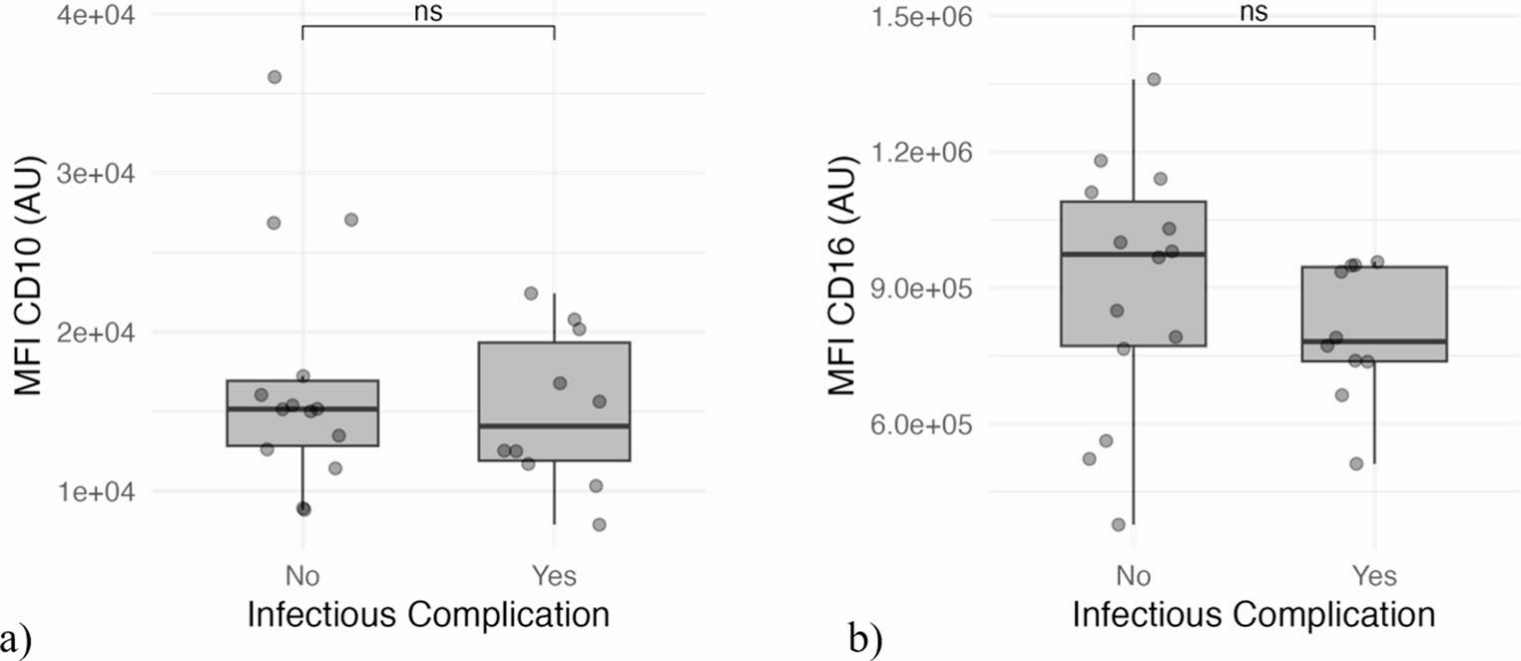
**a**) Boxplot-diagram comparing expression of CD10 in groups with and without infectious complications. **b**) Boxplot-diagram comparing expression of CD16 in groups with and without infectious complications

Ten out of twenty-four patients developed in-hospital infectious complications. The most common complication was pneumonia (five patients). Patients with infectious complications had lower mean expression levels of CD10 and CD16 on neutrophils than those without. However, these differences were not statistically significant (*p* = 0.71 and *p* = 0.11, respectively) (Fig. 3).

### Correlation of CD10 and CD16 with leukocyte-count

**Fig. 4:**
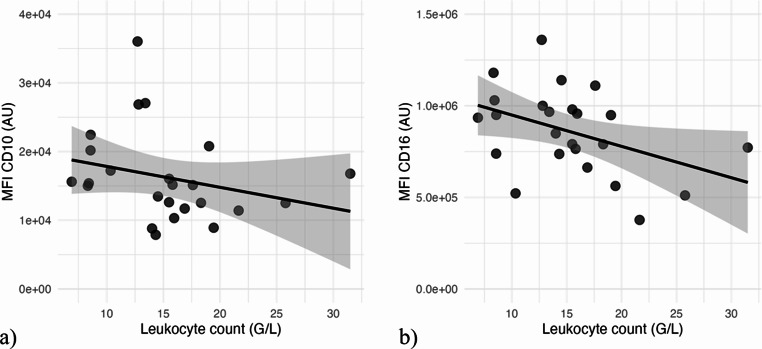
**a**) Diagram with correlation between leukocyte count at admission (×10⁹/L) and MFI CD10. **b**) Diagram with correlation between leukocyte count at admission (×10⁹/L) and MFI CD16

Leukocyte counts at admission ranged from 6.9 to 31.5 G/L, with a mean of 15.2 G/L and a standard deviation of 5.8 G/L. Spearman’s rank correlation revealed a moderate negative correlation between neutrophil-CD16 expression and leukocyte count (−0.448, *p* = 0.029) (Fig. 4b) as well between neutrophil CD10 expression and leukocyte count (−0.407, *p* = 0.0495) (Fig. 4a).

## Discussion

The current study underlines previously described [7, 10] early alterations in the systemic neutrophilic phenotype, and more specifically homogenous insult-related changes in the expression levels of CD10 and CD16 on circulating neutrophils after severe trauma. More interestingly, this study reveals that systemic neutrophil CD10 and CD16 expression levels upon admission correlate with elevated lactate levels 8 h post-injury in severe polytrauma. Lower overall expression of CD10 and CD16 on neutrophils has previously been identified and suggests either a concurrent decrease in both receptors on regular circulating neutrophils or, alternatively, a population-specific complementary shift. The latter has been suggested by Orr et al. and requires further research [5]. 

In the current study, instant shifts in neutrophil CD10 and CD16 receptor dynamics were further seen in parallel with the occurrence of posttraumatic leukocytosis. These observations suggest an influx of immature neutrophils from the bone marrow into the circulation and aligns with previous clinical studies demonstrating post-traumatic neutrophil mobilization and immune dysregulation, which may contribute to increased susceptibility to secondary infections [10, 13]. Furthermore, as systemic immune regulation demands high levels of energy consumption, it is tempting to speculate that this may be a relevant factor contributing to the pathophysiology of complicated trauma. Especially given the recently identified importance of mitochondrial disfunction upon trauma [14]. The interplay between changes of neutrophil cell surface expression levels and later deterioration of metabolic hemostasis, reflected by higher lactate levels, however, is currently unclear.

CD16, also known as Fcγ receptor III (FcγRs), is a low-affinity Fc receptor involved in neutrophil-mediated immune responses [15]. Since CD16 is predominantly expressed on mature neutrophils, its reduction suggests the presence of immature neutrophils, which may have an altered functional capacity [6, 16, 17]. Several studies in both human and porcine models, have reported a homogeneous decrease in neutrophil-CD16 expression in the first 24 h after trauma [18, 19]. The current study on post-insult circulatory neutrophil receptor dynamics after trauma, displays similar effects to the previous investigations.

CD10, also known as neutral endopeptidase, has been established as a marker to differentiate mature from immature neutrophils [5, 20]. Studies on CD10 after trauma are rare [10]. One study found a significant decrease in CD10 expression on circulating neutrophils after burn injuries [21]. These findings are in accordance with our data and it is likely, that if immature granulocytes are released from the bone marrow into blood, decreased overall expression levels of CD10 expression on blood neutrophils are seen. In the current study systemic neutrophil expression levels of CD10 were also significantly decreased in trauma, compared to healthy controls.

Serum lactate is a routine marker to describe metabolic status and guide treatment in severely injured patients. It reflects the extent of tissue hypoxia and increased anaerobic metabolism due to hemorrhagic shock or impaired perfusion. Elevated serum lactate concentrations have been associated with increased risk for infectious complications and higher mortality rates [22]. Studies showed that especially the lactate concentration in the first hours after insult as well as inappropriate lactate clearance are relevant predictors for mortality and complications, while lactate levels at admission have less predictive value [22–24]. The current study focused on 8 h lactate levels as this period reflects both, the impact of trauma (initial insult) and response to standardized trauma bay care, including the patient’s capacity to clear lactate [24]. The generally moderate lactate levels in our cohort likely reflect the effect of early and aggressive resuscitative treatment prior to the 8-hour measurement.

Given this time frame, identifying early prognostic markers for the most severely injured trauma patients is necessary. This study demonstrates a negative correlation between systemic neutrophil CD10 and CD16 expression and 8 h lactate levels, suggesting that neutrophil dysregulation on admission indicates the occurrence of metabolic deterioration. Moreover, the current study may imply a biological phenomenon that could explain an interesting interplay between early massive intercompartmental neutrophil movements and metabolic dysregulation in critically ill patients. Specifically, it is not unlikely that neutrophil migration processes actually contribute to the development of metabolic stress due to increased energy consumption. Conversely, metabolic stress itself may further modulate neutrophil behavior, perpetuating a vicious cycle of immune dysregulation and metabolic disturbance.

In patients who developed infectious in-hospital complications, CD10 and CD16 expression levels on circulatory neutrophils were lower than in those without infections. Although statistical significance was likely not achieved due to the limited sample size, this seems to be in accordance with the small amount of existing literature [10, 13, 25]. A recent study identified increased levels of CD16^dim^/CD62^bright^ neutrophils after polytrauma and named them a risk factor for infectious complications [10]. More multicenter studies are needed to prospectively validate these findings, especially in the most endangered trauma patients, namely the severely injured.

## Conclusion

Changes in the neutrophil CD10/CD16 profile, that are linked to leukocytosis, precede metabolic dysregulation. A better understanding of the early neutrophil receptor profile may form the basis of future immune-monitoring and guide treatment decisions. Furthermore, profound neutrophil shifts in the most life-endangered trauma patients likely result in increased energy demands and may therefore contribute to the development of metabolic deterioration in this subgroup.

## Data Availability

The datasets used and/or analyzed during the current study are available from the corresponding author on reasonable request.
